# Coronavirus Disease 2019 Safety Measures for Sustainable Tourism: The Mediating Effect of Tourist Trust

**DOI:** 10.3389/fpsyg.2022.784773

**Published:** 2022-02-28

**Authors:** Muddassar Sarfraz, Mohsin Raza, Rimsha Khalid, Larisa Ivascu, Gadah Albasher, Ilknur Ozturk

**Affiliations:** ^1^College of International Students, Wuxi University, Wuxi, China; ^2^Department of Management Sciences, Phuket Rajabhat University, Phuket, Thailand; ^3^Department of Business and Management, Limkokwing University of Creative Technology, Cyberjaya, Malaysia; ^4^Department of Management, Faculty of Management in Production and Transportation, Politehnica University of Timişoara, Timişoara, Romania; ^5^Research Center in Engineering and Management, Politehnica University of Timişoara, Timişoara, Romania; ^6^Department of Zoology, College of Science, King Saud University, Riyadh, Saudi Arabia; ^7^Higher Vocational School, Cag University, Mersin, Turkey

**Keywords:** tourist safety measures, tourist psychology, tourist trust, COVID-19, sustainability, tourism destination choice

## Abstract

Coronavirus disease 2019 (COVID-19) pandemic is continuing to have severe effects on tourism-related industries, as safety precautions have become essential to follow. Based on this, this study aims to explore the role of perceptions of the tourist of safety in tourism destination choice with the mediating effect of tourist trust (TT) in the context of the Chinese tourism sector. In addition, this study considers improvements to safety measures for sustainable tourism and the benefits of the technology transformation in the travel industry because of COVID-19. For this study, a quantitative approach was used, and data were collected through convenient sampling. The questionnaire was measured on a 5-point Likert scale, and a cross-sectional approach was adopted for data analysis. The findings of this study show that the effect of the perceived safety of the social environment, perceived safety of facility and equipment elements, perceived safety of human elements, perceived safety of management elements, and perceived safety of natural environments is significant and positive on the tourist destination choice (TDC). In addition, TT is a significant mediator between these elements and TDC. Furthermore, this study concluded that COVID-19 had increased travel anxiety, with particularly negative effects on the Chinese tourism sector, but that the adoption of perceived safety measures could be beneficial in regaining TT for traveling, eventually giving tourists confidence in choosing their traveling destination.

## Introduction

Disastrous economic impacts of coronavirus disease 2019 (COVID-19) are now spread worldwide, and China is not exempted from those impacts. Because of the effects of COVID-19, economy of China has experienced a “shutdown” (Liu and Hu, 2020). Similarly, Chinese value-added industries have declined by 13.5% as a result of COVID-19, the production index of the service industry declined by 13.05, investment in fixed assets dropped by 24.5%, and RMB exports declined by 15.9%. At last, total social consumer goods retail sales have fallen by 20.5% (Liu and Hu, 2020). Thus, China has paid a high-economic price to avoid and manage the devastating effects of COVID-19. In this way, economy of China has endured insecurity and uncertainty, particularly in the tourism sector. Tourism industry of China was the first to bear the brunt of the devastation of COVID-19 (Hao et al., 2020). Before the pandemic broke out, the tourism of China serves as a backbone in the economy of the country. Based on the report of the World Economic Forum, the total contribution of tourism industry of China is almost 10% of the total gross domestic product of the world (Faus, 2020). China is an attractive destination and a hub of incoming and outgoing tourists in the global tourism market. Journeys inside the country add a major portion to the revenue of the country, which previously reached CN¥ 5,128 billion annually. However, in this challenging time, tourism sector of China has been the most severely affected of all the industries (Ayittey et al., 2020). In the early days of the crisis, adverse economic effects caused by COVID-19 extended beyond Hubei borders (where the virus originated), with the closure of more than 70,000 theaters and major airlines, significantly disrupting tourism activities. This disruption soon spread globally; by using an estimated modeling approach, Bloomberg economists anticipated a 0.42% decline in the GDP of the world in the first 3 months of 2020, resulting from expected economic losses because of many countries fighting outbreaks (Ayittey et al., 2020).

In addition, tourism in China has also been affected seriously due to the global reporting of mass media on the origins of virus in China. According to Zheng et al. (2020) and Xie et al. (2021), due to the negative reporting of the mass media, the many denoted COVID-19 as the “China virus,” discouraging people from planning tours to China and negatively impacting attitudes to those traveling from there. As a result, on February 7, 2020 Dass and McDermott (2020) estimated about a US$22 billion reduction in spending of outbound tourists, with inbound tourists reducing to just 9 million. Chinese departures in 2020 have been limited to 7–25 million, affecting many destinations across the country. Furthermore, all the guided tours throughout China conducted by the tourism agencies have been canceled, including Beijing, where 13,525 tours were canceled, restraining 242,000 travelers for tourism (Nan, 2020). To date, almost 130 nations have barred citizens of China from entering their borders by enacting proportional measures.

As a result, China has undertaken a countrywide all-inclusive approach to fight the spread of the novel coronavirus (Liu et al., 2020). The Chinese government has claimed to have controlled 90% of the cases, and it has also hoped to restart domestic tourism in the future. The government is encouraging the sustainable tourism-related industry to restart their businesses for international tourists by strictly considering the safety measures against COVID-19 (Foon, 2020). Technology is an essential element to develop the sustainable tourism industry in COVID-19 situation. Globally, the technological factor has become progressively involved in businesses including those in the travel industry (Khalid et al., 2021). It is vital to involve the tour operator in using technology to create a sustainable tourism industry in China (Qiu et al., 2020). Because of the pandemic, information technology has become more widely available for access to useful resources and comprehensive, adaptable, and value-added solutions to some problems in daily lives of humans, related to work, travel, recreation, business, and government. Technology has played a significant role in developing sustainable tourism overall in the world (Bae and Chang, 2021).

Meanwhile, safety issues have been treated as essential factors for tourists (Woosnam et al., 2015; Hassan and Soliman, 2021). Furthermore, information technology seems very effective in preventing transmission on both a local and international scale (e.g., tourist inspections, cases and contact assessments, online learning, etc.). Tourists always consider specific safety measures while traveling, such as avoiding walking in remote areas, having an awareness of the environment, etc. During COVID-19 pandemic, tourists have become more conscious of adopting precautionary measures (Godovykh et al., 2021; Perić et al., 2021). For tourists, the experience of good safety measures plays a very important role in building trust. For example, tourists from various countries have different views of safety in terms of socioeconomic, environmental, societal, political, and other potential risk factors (Seabra et al., 2013).

To define this, Xie et al. (2021) suggested the following five dimensions of the tourist perceived safety: (1) perceived safety of facility and equipment elements (PSFEs), which refers to the perception of the facilities, safety assessments, and equipment within destinations; (2) perceived safety of human elements (PSHEs), meaning perceptions of individual behavior, and safety assessments in a tourism context; (3) perceived safety of management elements (PSMEs), which refers to the perception of tourism safety management policies, safety assessments and actions, and related aspects at the organizational or managerial levels; (4) perceived safety of natural environment (PSNE) represents safety or sustainability of natural elements such as water reservoirs, mountains, trees, animals, and atmosphere that make up the natural ecosystem; and (5) perceived safety of social environment (PSSE), meaning perceptions of the environmental factors and safety assessments of destinations. As such, provision of solutions to address these factors can play a crucial role in developing trust of the tourists (Jensen and Blichfeldt, 2009; Pagliara et al., 2021). This is pivotal, as trust of the tourists might affect tourist destinations choices (Eitzinger and Wiedemann, 2008; Artigas et al., 2017).

Here, we contribute to the destination choice literature by linking the perceived safety factor to trust and then to tourist destination choice (TDC) with technological transformation. To our knowledge, no attempt has been made thus far to link tourist perceived safety and its dimension with tourist trust (TT) and choice of destination. Because of the negative image caused by COVID-19, China needs to establish and create tourist perceptions of healthy tourism in China (Xie et al., 2021). Thus, in this regard, the main objective of the study is to examine the role of tourist perceived safety on TDC with the mediating role of TT.

## Theoretical Background and Hypothesis Development

### Tourist Destination Choice

The concept of TDC is based on the rational tourist behavior, which comprises understanding the destination choice and eliminating the alternatives because of some non-psychological and socio-psychological aspects (Saito and Strehlau, 2018). It represents the aspirations of the latter for a certain action in a specific situation and could be implemented as the possibility of intention (Lai et al., 2018; Pan et al., 2021). When there is an incentive to have an intention, purpose leads to actions; it can provide the greatest indicator of behavior (Fishbein and Ajzen, 1975). Researchers (Kim and Richardson, 2003) suggest that the picture of a destination influences tourism-related attitudes by reinforcing current attitudes, forming new attitudes, or changing attitudes. Likewise, Phillips and Jang (2008) have found that perception of a tourist of a destination can affect their attitude toward such a destination.

On the contrary, according to Hsu et al. (2009), various variables affect preference of destination of the tourists, including cultural accessibility, geographical location, natural encounters, personal protection, and entertainment attractions. When deciding where to go, safety and protection were regularly rated as the least significant aspect (Lu, 2019). This was expressed in the hospitality and tourism study, which focused on protecting tourist destination selection/choice. Residents, elected officials, and representatives of the restaurant industry in New Orleans increasingly voiced anxiety about community protection. They witnessed a decline in tourism market volume because of the growth rate of the city of violent crime (Sirakaya et al., 1997). Lu (2019) investigated whether the extent of perceived safety and support would influence the final decision to holiday at a specific location.

### Tourist Perceived Safety

Tourist perceptions of safety is focused on a holistic assessment of security of the destination, including the expected level of safety for the physical self and personal property. In some way, TPS is perceived safety picture of a destination. Hence, TPS has been described as an influencing factor affecting travel decision-making procedures of tourists, significantly affecting satisfaction, confidence/trust, and revisit intentions. Furthermore, tourists develop safety judgments based on the variety of safety sources to which they are attracted (Seabra et al., 2013; Su et al., 2021); e.g., contacts between tourists and residents, natural environments, facilities and equipment, public security systems, and so on. Hence, this research conceptualized TPS as a multidimensional construct consisting of humans, facilities and equipment, environments, and management and the dimensions of tourist perceived safety.

### Perceived Safety of Social Environment

According to Xie et al. (2021), PSSE means perceptions of environmental factors and safety assessments of destinations. They are the well-articulated optimistic environmental factors that support regular tourism operation. The natural world and the social-cultural background are divided into two sections. While their features and origins vary, information regarding protection issues in natural ecosystems and individual cultures positively affects TDC.

Politics, economies, history, and local populations also contribute to the socio-cultural framework, an urban structure formed through human social practices. Political unrest (Gartner and Shen, 1992), economic instability (Alegre et al., 2013), and foreign terrorism form travel protection perceptions at a macro stage, increasing the psychological pressure on visitors in unknown environments (Jenkin, 2006). People who have already arrived at their destinations might be a little more worried about certain threats and shape individualized safety expectations based on local knowledge about orderliness, disputes, and food safety. Tourists, according to Ryan (2013), are “displaced” people who ignore their usual responsibilities and are more vulnerable in new environments (Eitzinger and Wiedemann, 2008). Theft, burglary, misunderstandings, or violations of road laws resulting in traffic accidents can occur. As a result, they are more worried regarding public safety and travel statistics (Choocharukul and Sriroongvikrai, 2017). In terms of travel options and interactions, the consistency of food service and the selection of various rates play a significant role (Sheldon and Fox, 1988). Food protection and local hygiene practices are important factors in destination selection and safety expectations (Lee et al., 2019). In addition, when shopping, another important aspect of customer experience assessments is safety (Yuksel, 2004). Therefore, this research hypothesizes:

H1: There is a positive effect of PSSE on Tourism Trust.

### Perceived Safety of Facility and Equipment Elements

The PSFEs refers to perception of the facilities, safety assessments, and equipment within destinations (Xie et al., 2021). They are a demonstrable group of constructive facility and equipment factors that help to promote regular tourism operation. Destination services and equipment should be reviewed, checked, maintained, and upgraded regularly. Furthermore, the proper matching of products and services to individual visitors improves protection (Bentley et al., 2001).

The reliable and secure running of equipment and facilities is critical for visitors who have already arrived at their venue. People are worried about the safety conditions of specific services and appliances in hotels. When choosing a hotel, fire suppression devices, safety lighting systems, automatic door controls, food protection, and safety checking are also critical considerations (Chan and Lam, 2013).

Furthermore, people with disabilities have higher safety standards and preferences regarding mobility, assistive technology, and specialized facilities (Tutuncu, 2017). High-risk activities such as whitewater rafting, mountain and rock climbing, hang gliding, and skydiving require safety. These are heavily reliant on the protective success of vehicles and services and those that accompany them in adventure travel. Mistakes in these areas result in injuries, giving destinations and providers a bad reputation for protection. Adding risk alert devices improves destination and employee protection expectations by efficiently communicating the degrees of threat and acceptable activities (Rittichainuwat, 2013). Therefore, this research hypothesizes:

H2: There is a positive effect of PSFEs on TT.

### Perceived Safety of Human Elements

The PSHEs refers to perceptions of individual behavior and safety assessments in the tourism context (Xie et al., 2021). It is a measurable collection of optimistic human characteristics linked to regular tourism behavior. This involves the behavior of tour guides, tourist destinations, locals, fellow travelers, and tourists. Differences in gender, age, perceived risk, risk-related capabilities, and prior experience can cause differences in the handling of destination safety information (Fourie et al., 2020). Misconduct, e.g., is a frequent occurrence in travel groups. This might contribute to verbal and physical abuse, bribery, fraud, and robbery, all of which interrupt source and a destination and degrade travel experiences, often resulting in accidents or the loss of personal assets (Tsaur et al., 2019). Furthermore, low service quality, high perishability, and inseparability of facilities worsen the dangers of tourism (Qin et al., 2021). In addition, relational unity (positive feelings for one another) of the citizens reinforces the sense of protection (Woosnam et al., 2015). Therefore, this research hypothesizes:

H3: There is a positive effect of PSHEs on Tourism Trust.

### Perceived Safety of Management Elements

The PSMEs represent the perception of tourism safety management policies, safety assessments and actions, and related aspects at the organizational or managerial levels (Xie et al., 2021). They are examples of good management that promotes regular tourism events. Moreover, aspects such as protection infrastructure elements (Becken and Hughey, 2013) (security institutions, evacuation plans) and also behavioral safety elements (safety records, police, as well as emergency rescue services) need to be implemented by destinations (Gurtner, 2016). These management structures can be encountered at the destination and could be affecting views of people of protection of management. For example, many nations, including the United Kingdom, and Spain, have enacted safety laws to prevent accidents and deaths associated with diving tourism and coastal events (Coxon, 2006). Government protection programs, such as alerts, crisis prevention, and disaster response strategies, improve perceptions of safety of the tourists and help boost visitor interest during or after a crisis (Gurtner, 2016). According to Rittichainuwat (2013), inbound views of tourists of beach protection are based on the provision of an emergency response plan and a tsunami evacuation scheme (Rittichainuwat, 2013). In addition, several famous tourist attractions (such as South Korea and Turkey) have set up separate tourism policy divisions to comply with security concerns. Tourist protection standards and views of destinations are heavily influenced by police culture or service quality (Sharma et al., 2016). In general, tourism business protection monitoring such as preflight safety alerts on airlines (Lee et al., 2018), hotel security, safety services (Chan and Lam, 2013), and visitor attraction disaster management strategies (Rittichainuwat, 2013), enhances destination safety expectations. As a result, PSME explain the safety standards and attitudes of corporate safety activities of destinations. Therefore, this research hypothesizes:

H4: There is a positive effect of PSMEs on Tourism Trust.

### Perceived Safety of Natural Environments

The PSNEs relates to physical elements such as water reservoirs, mountains, trees, animals, and the atmosphere that make up the natural ecosystem (Xie et al., 2021). They serve as an attraction basis for resort construction and contain the knowledge to determine safety overall risk. Places to visit with high-quality and iconic natural environments such as pristine beaches, breathtaking scenery, and unique flora and fauna (Hosany et al., 2007), enable tourists to feel self-satisfied and comfortable, and enjoy themselves. Volcanoes and previous natural hazard sites are examples of areas vulnerable to natural disasters. People develop their first safety evaluations about these locations based on news reports and other evidence available, and their expectations of safety are shaped as a result (Rittichainuwat, 2013; Poon and Koay, 2021). Climate-sensitive locations are diverse and complex (Scott and Lemieux, 2010), and can influence experiences of the travelers differently. Extreme weather events (rising ocean levels, high temperatures) are becoming more common because of the climate change, which will have a detrimental effect on travel plans and expectations of comfort and protection (Hübner and Gössling, 2012; Robina-Ramírez et al., 2021). Global warming is a critical factor that presents threats to visitors and resorts so it must be addressed. Furthermore, destinations in or around desert areas pose a greater risk of damage and personal injuries, with individuals becoming more prone to suffer from heatstroke, hypothermia, or being lost (Eitzinger and Wiedemann, 2008). Therefore, this research hypothesizes:

H5: There is a positive effect of PSNEs on Tourism Trust.

### Tourism Trust

Trust has been described in the tourism literature as the dependability and credibility of important components associated with perceptions of destinations of the tourists (Artigas et al., 2017). Related to tourism literature, the concept of trust started to be examined in the late 1990s (Crotts et al., 1998). Still, most of the experiments were collaborative and use trust concepts from sociology and psychology (Morgan and Hunt, 1994). With recent cases of trust crises in the tourism industry such as unfair or unethical treatment by travel agencies (Chang, 2014) and hotels failing to deliver the premium service promised (Lien et al., 2015), to mention a few, the definition of TT has now become a major topic among academics and practitioners, and further investigations have been conducted.

Tourist faith/trust is intricately linked to profile of a destination. The brand of a tourist destination is seriously harmed if it is seen as distrustful, rendering it dangerous to visit. It is fair to assume that visitors who are dissatisfied with the destinations because of the experience they see and discover at the place will be guided by these experiences when building their trust structure (Williams and Baláž, 2013). Consequently, trust crisis events cause visitors to be concerned about their safety and potential dangers, and a negative impression of the destination is created in their perceptions (Chew and Jahari, 2014).

Many researchers in the tourism sector have shown that TT/confidence has a huge impact on destination choice of a traveler. For instance, according to Lepp et al. (2011), many of the documented travel risks in Africa, including a high-murder rate, unsafe food, and unwelcoming hosts, have severely harmed it as a destination choice. Furthermore, as Chew and Jahari (2014) demonstrated, Fukushima nuclear disaster of Japan in 2011 caused concerns of radioactive poisoning and polluted food and air, negatively impacting reputation of the country as a tourist destination. Put simply, destination trust ensures that tourists who want to visit a specific destination can receive services that are straightforward, dependable, risk-free, and hassle-free (Roodurmun and Juwaheer, 2010). Destination trust/confidence, according to Abubakar and Ilkan (2016), relates to readiness of a visitor to trust capacity of a tour destination to fulfill its advertised functions.

Williams and Baláž (2021) contend that trust of tourists’ in safety management of a tourist location is essential when estimating vacation risks. When tourists trust safety and protection of a destination point, their perceived danger could be lower. Negative knowledge about a visitor destination’s protection has a more significant influence on mistrust than positive information about travel safety has on trust, according to empirical findings for the asymmetry theory of trust specifically, and for a skepticism bias concerning uncertainty knowledge in general (Eitzinger and Wiedemann, 2008). In other words, disclosing the absence of appropriate security measures and circumstances (negative information) can lead to a far higher level of mistrust in safety management of a destination country than disclosing the existence of acceptable safety precautions and circumstances (positive information).

Therefore, this study has the following proposition:

H6: There is a positive effect of TT on TDC.

H7: There is a mediating effect of Tourism Trust between the relationship of PSSE and TDC.

H8: There is a mediating effect of Tourism Trust between the relationship of PSFEs and TDC.

H9: There is a mediating effect of Tourism Trust between the relationship of PSHEs and TDC.

H10: There is a mediating effect of Tourism Trust between the relationship of PSMEs and TDC.

H11: There is a mediating effect of Tourism Trust between the relationship of PSNEs and TDC.

**FIGURE 1:**
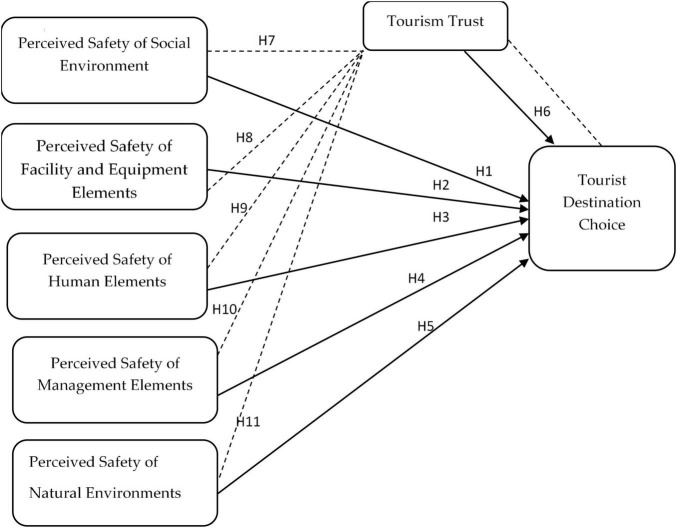
Conceptual model.

Figure 1 shows study conceptual framework.

## Research Methodology

Data for this research was gathered online by email from early April to late July 2021, a period during which there was a significant increase in COVID-19 cases in China. Therefore, safety views of the respondents toward TDC remained consistent for months. Potential tourists were asked to complete the survey to follow government health guidelines and alleviate their concerns about the health risks connecting with personal interaction. The data were collected using a convenience sample technique. Convenience sampling is a non-probability or non-random sampling where members of the target population meet certain practical criteria, such as easy accessibility, geographical proximity, availability at a given time, or the willingness to participate are included for the study (Taherdoost, 2016). For the collection of data, a standard operating procedure was followed strictly. The researcher and his assistant wore masks, kept a safe distance while interacting with tourists, and collected data from a variety of locations, including lakes (36%), beaches (29%), and other sights (historic sites, mountains; 35%). Occasionally, if they had any query concerning the questions, it would be resolved. Two hundred and seventy tourists participated in the study; however, 11 questionnaires were excluded owing to inadequate information. This study is cross-sectional and quantitative. The participants were asked to recall their recent weekend visit to a tourism destination. There were both male and female respondents. The 5-point Likert was used to obtain the responses to analyze the data. Furthermore, partial least squares structural equation modeling [PLS (SEM)] was used to analyze the data.

### Measurement

Perceived safety of human elements has five items with 0.862 Cronbach’α, PSFEs has five items with Cronbach’α = 0.882, PSSE has five items with Cronbach’α = 0.871, PSNEs has three items with Cronbach’α = 0.865 and PSMEs also has five items with Cronbach’α = 0.852 all were developed by Zhang et al. (2020). TT has six items with 0.87 Cronbach’α developed by Bambauer-Sachse and Mangold (2011). Furthermore, TDC has six items with Cronbach’α 0.87 developed by Bambauer-Sachse and Mangold (2011).

## Data Analysis

This analysis of the study is divided into two parts. The measurement model assessment is the first part. The structural model evaluation, of which hypotheses were evaluated, is the second component. A measurement model measures the latent variables or composite variables, while the structural equation model tests the all hypothesis based on path analysis (Hoyle, 2011). In addition, the *R*^2^ value, as well as model quality, are discussed in this section.

### Measurement Model Assessment

**FIGURE 2:**
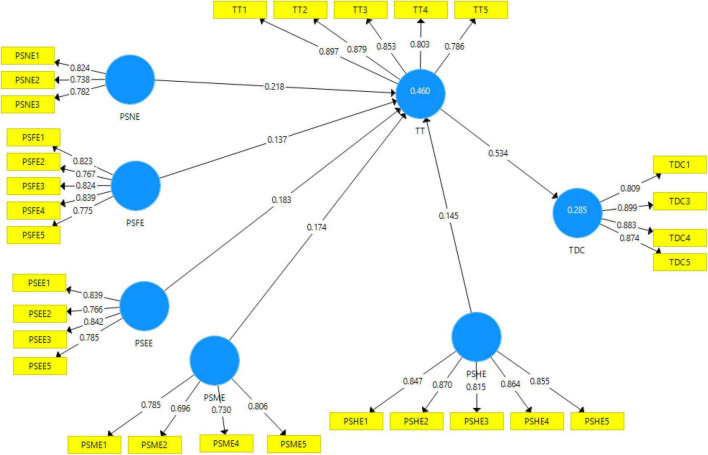
Measurement assessment model.

Factor loading, composite reliability CR, and average variance extracted (AVE) were used in the first part of the study (Hair et al., 2016). Factor lodgings will be greater than 0.5, and all items below 0.5 should be eliminated (Hair et al., 2016). The rule of thumb proposed by George and Mallery (2003) is that meaning of 0.7 is appropriate. In addition, the CR will be greater than 0.7. Moreover, AVE must be greater than or equivalent to 0.5 to obtain convergent validity and internal consistency. The findings of the calculation model evaluation are seen in Table 1. All the values are well beyond reasonable limits. Factor loading is greater than 0.7, and CR is also greater than 0.7. In addition, AVE is greater than 0.5, indicating convergent validity. Furthermore, Figure 2 shows the pictorial presentation of measurement assessment model (Figure 2).

**TABLE 1:** Internal consistency.

Constructs	Cronbach’s alpha	rho_A	Composite reliability	Average variance extracted (AVE)
PSSE	0.825	0.835	0.883	0.654
PSFE	0.866	0.874	0.903	0.65
PSHE	0.905	0.91	0.929	0.723
PSME	0.759	0.776	0.841	0.571
PSNE	0.702	0.747	0.825	0.612
TDC	0.889	0.893	0.924	0.751
TT	0.899	0.903	0.926	0.714

The square root of AVE and cross-loadings is used to achieve discriminant validity. Following orders of Chin, cross-loadings were investigated (Chin, 1998). Following orders of Fornell and Larcker (1981), the square root of average variance was extracted and analyzed. Table 2 shows the discriminant validity between constructs and Table 3 displays the variance explained (*R*^2^) in endogenous variables. In addition to hypotheses testing, the value of *R*^2^ of TDC is 0.285 is explained, and TT is IVs explained 48%. The value of *R* adjusted of TDC is 0.283, and TT is 0.453.

**TABLE 2:** Discriminate validity.

	PSEE	PSFE	PSHE	PSME	PSNE	TDC	TT
PSEE	0.809						
PSFE	0.793	0.806					
PSHE	0.487	0.433	0.851				
PSME	0.507	0.458	0.425	0.756			
PSNE	0.493	0.433	0.487	0.71	0.782		
TDC	0.477	0.457	0.831	0.46	0.503	0.867	
TT	0.559	0.52	0.474	0.546	0.562	0.534	0.845

**TABLE 3:** *R*^2^ and adjusted *R*^2^.

Constructs	*R* Square	*R* Square adjusted
TDC	0.285	0.283
TT	0.46	0.453

### Structural Model Assessment

**FIGURE 3:**
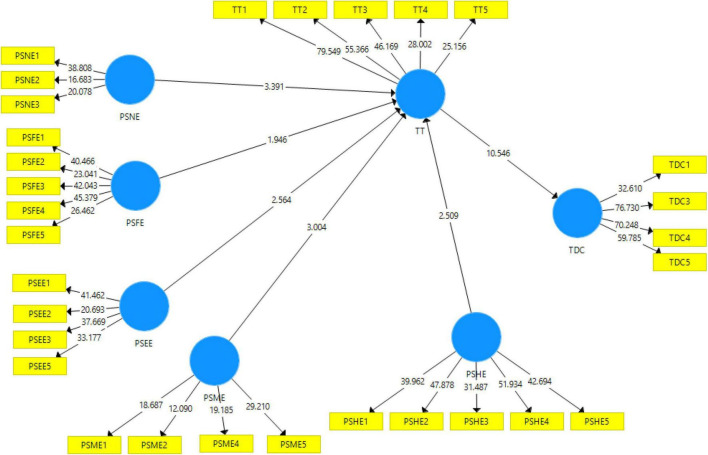
Structural model.

The measurement of hypotheses was the focus of the second part of the study. Direct and mediation hypotheses are used. First, as seen in Table 4, direct hypotheses have been examined. A *p*-value of 1.96 was used to endorse or refute the hypotheses. Both partnerships with a *t*-value less than 1.96 would be refused, whereas any with a *t*-value greater than 1.96 (*t*-value > 1.96) should be approved. Table 4 indicates that all of the relationships have a *t*-value greater than 1.96, indicating meaningful. As a result, the direct hypotheses have been accepted. H2, on the other hand, is rejected. Figure 3 shows structural model.

**TABLE 4:** Direct relationship.

Hypothesis	Relationship	Original sample (O)	Std. dev.	*T* Statistics	*P* Values	Decision
H1	PSSE -> TT	0.183	0.072	2.547	0.011	Accepted
H2	PSFE -> TT	0.137	0.071	1.928	0.054	Rejected
H3	PSHE -> TT	0.145	0.057	2.525	0.012	Accepted
H4	PSME -> TT	0.174	0.058	2.981	0.003	Accepted
H5	PSNE -> TT	0.218	0.063	3.476	0.001	Accepted
H6	TT -> TDC	0.534	0.05	10.619	0	Accepted

This study found a significant result between PSEE and TT (β = 0183, *t* = 2.547, *p* = 0.011). So, H1 is accepted. Hypothesis 2 predicted that PSFE is positively related to TT. Table 4 established a non-significant and positive relationship between PSFE and TT (β = 0.137, *t* = 1.928 < 1.96, *p* = 0.054 > 0.05) rejecting hypothesis 2. Hypothesis 3 predicted that PSHE is positively related to TT. Table 4 established a significant and positive relationship between PSHE and TT (β = 0.145, *t* = 2.525 > 1.96, *p* = 0.012 < 0.05) accepting hypothesis 3. Hypothesis 4 predicted that PSME is positively related to TT. Table 4 established a significant and positive relationship between PSME and TT (β = 0.174, *t* = 2.981 > 1.96, *p* = 0.003 < 0.05) accepting hypothesis 4. Hypothesis 5 predicted that PSNE is positively related to TT. Table 4 established a significant and positive relationship between PSNE and TT (β = 0.218, *t* = 3.476 > 1.96, *p* = 0.001 < 0.05) accepting hypothesis 5. Hypothesis 6 predicted that TT is positively related to TDC. Table 4 established a significant and positive relationship between TT and TDC (β = 0.534, *t* = 10.619 > 1.96, *p* = 0.000 < 0.05), accepting hypothesis 6.

In Table 5, hypothesis 7 predicts that the mediating effect of tourism trust between the relationship of PSSE and TDC is significant (β = 0.098, *t* = 2.480 > 1.96, *p* = 0.013 < 0.05), therefore, accepted hypothesis 7. Hypothesis 8 predicts that the mediating effect of tourism trust between the relationship of PSFE and TDC is non-significant (β = 0.073, *t* = 1.888 < 1.96, *p* = 0.059 > 0.05), therefore, rejected hypothesis 8. Hypothesis 9 predicts that the mediating effect of tourism trust between the relationship of PSHE and TDC is significant (β = 0.077, *t* = 2.147 > 1.96, *p* = 0.032 < 0.05); therefore, it accepted hypothesis 9. Hypothesis 10 predicts that the mediating effect of tourism trust between the relationship of PSME and TDC is significant (β = 0.093, *t* = 3.063 > 1.96, *p* = 0.002 < 0.05), therefore, accepted hypothesis 10. Hypothesis 11 predicts that the mediating effect of tourism trust between the relationship of PSNE and TDC is significant (β = 0.116, *t* = 3.204 > 1.96, *p* = 0.001 < 0.05), therefore, accepted hypothesis 11.

**TABLE 5:** Indirect relationship.

Hypothesis	Relationships	Original sample	Std dev.	*T* Statistics	*P* Values	Decision
H7	PSSE -> TT -> TDC	0.098	0.039	2.480	0.013	Accepted
H8	PSFE -> TT -> TDC	0.073	0.039	1.888	0.059	Rejected
H9	PSHE -> TT -> TDC	0.077	0.036	2.147	0.032	Accepted
H10	PSME -> TT -> TDC	0.093	0.03	3.063	0.002	Accepted
H11	PSNE -> TT -> TDC	0.116	0.036	3.204	0.001	Accepted

Finally, the quality of the model was observed through predictive relevance (*Q*^2^). The measurement is exchanged to goodness-of-fit. Chin (1998) argued that the value of *Q*^2^ should be above zero. Table 6 shows that the value of *Q*^2^ of TDC 0.201 > 0 and TT is 0.303 > 0.

**TABLE 6:** Predictive relevance.

Constructs	SSO	SSE	(*Q*^2^ = 1-SSE/SSO)
PSSE	1,560.00	1,560.00	
PSFE	1,950.00	1,950.00	
PSHE	1,950.00	1,950.00	
PSME	1,560.00	1,560.00	
PSNE	1,170.00	1,170.00	
TDC	1,560.00	1,246.61	0.201
TT	1,950.00	1,359.36	0.303

## Discussion

The main objective of the study is to examine the mediating effect of TT between perceived safety and TDC. This study found a significant result between PSSE and TT (β = 0183, *t* = 2.547, *p* = 0.011). So, H1 is accepted. The result shows that the natural environment and the social-cultural context create trust among the Chinese tourists (Lee et al., 2019). Hypothesis 2 predicted that PSFE is positively related to TT. Table 4 established a non-significant and positive relationship between PSFE and TT (β = 0.137, *t* = 1.928 < 1.96, *p* = 0.054 > 0.05) rejecting hypothesis 2. The result shows that equipment provided by management to the tourist is unsatisfactory that builds mistrust among tourists. Hypothesis 3 predicted that PSHE is positively related to TT. Table 4 established a significant and positive relationship between PSHE and TT (β = 0.145, *t* = 2.525 > 1.96, *p* = 0.012 < 0.05) accepting hypothesis 3. The result shows that safety assessments and perceptions behavior in tourism settings of the tourists are satisfactory and build trust, similar with Fourie et al. (2020). Hypothesis 4 predicted that PSME is positively related to TT. Table 4 established a significant and positive relationship between PSME and TT (β = 0.174, *t* = 2.981 > 1.96, *p* = 0.003 < 0.05) accepting hypothesis 4. The result shows that tourism safety management policies and actions of the managerial level are acceptable for Chinese tourists (Li et al., 2021). Hypothesis 5 predicted that PSNE is positively related to TT. Table 4 established a significant and positive relationship between PSNE and TT (β = 0.218, *t* = 3.476 > 1.96, *p* = 0.001 < 0.05), accepting hypothesis 5. The result shows that natural environments and physical resources are enough to produce trust among Chinese tourists, the results are similar with Robina-Ramírez et al. (2021). Hypothesis 6 predicted that TT is positively related to TDC. Table 4 established a significant and positive relationship between PSNE and TT (β = 0.534, *t* = 10.619 > 1.96, *p* = 0.000 < 0.05), accepting hypothesis 6. The results show that tourist has trust whatever he or she is given at the spot that factor builds the positive intention to visit that place (Sun et al., 2021). Hypothesis 7 predicts that the mediating effect of tourism trust between the relationship of PSEE and TDC is significant (β = 0.098, *t* = 2.480 > 1.96, *p* = 0.013 < 0.05), therefore, accepted hypothesis 7. The result shows that both the natural environment and the cultural perspective decide to visit that place through strong trust among Chinese tourists (Wu et al., 2021). Hypothesis 8 predicts that the mediating effect of tourism trust between the relationship of PSFE and TDC is non-significant (β = 0.073, *t* = 1.888 < 1.96, *p* = 0.059 > 0.05), therefore, rejected the hypothesis 8. The result shows that equipment of the management is unsatisfactory, that builds mistrust among tourists and decides not to go to that place. Hypothesis 9 predicts that the mediating effect of tourism trust between the relationship of PSHE and TDC is significant (β = 0.077, *t* = 2.147 > 1.96, *p* = 0.032 < 0.05); therefore, it accepted hypothesis 9. The result shows that safety assessments and perceptions of behavior of tourists in tourism settings are quite satisfactory, building trust in visiting that place (Demir, 2021). Hypothesis 10 predicts that the mediating effect of tourism trust between the relationship of PSME and TDC is significant (β = 0.093, *t* = 3.063 > 1.96, *p* = 0.002 < 0.05), therefore, accepted hypothesis 10. The result shows that tourism safety management policies and actions of managerial level are acceptable for Chinese tourists that build trust in deciding to visit that place (Yung et al., 2021). Hypothesis 11 predicts that the mediating effect of tourism trust between the relationship of PSNE and TDC is significant (β = 0.116, *t* = 3.204 > 1.96, *p* = 0.001 < 0.05), therefore, accepted hypothesis 11. The result shows that natural environments and physical resources are enough to produce trust among Chinese tourists in deciding to visit that place (Geng et al., 2021).

## Conclusion

The results of this study conclude that there is a significant relation between PSSE and Tourism Trust, PSHEs and Tourism Trust, PSMEs and Tourism Trust, PSNEs and Tourism Trust, TT, and TDC. Meanwhile, the study indicates that there is an insignificant relation between PSFEs and TT. Moreover, the study shows that there is a significant mediating effect of Tourism Trust between PSSE and TDC, PSHEs and TDC, PSMEs and TDC, PSNEs and TDC. However, the study found that there is an insignificant mediating effect of Tourism Trust between PSFEs and TDC.

### Theoretical Contribution

This research has examined the role of tourist perceived safety in TDC along with the mediating role of TT. This study also examined the factors of TT, which is a useful contribution to the literature on tourist destinations. This study offers perceived safety factors of the tourists as related to the technological element as a theoretical contribution. These factors enhance the literature of perceived safety toward tourism destination choice. Furthermore, the mediating variable TT plays a pivotal role to explain the relationship between perceived safety factors and TDC.

### Practical Implications

This study has many practical consequences. First, destination management organizations (DMOs) can use the TPS scale to understand safety perspectives better and to create customized strategies to sustain a healthy tourist destination environment based on individual dimensions. Technological advancement is contributing to tourism; in addition, DMOs must consider the actions of a tourist guide, locals, tourism operators, and even tourists themselves when managing human safety with technology. Furthermore, DMOs can improve the relationship between tourists and these safety elements, thus, increasing trust of people in new environments and traveling safely.

### Limitations and Future Research

This study has some limitations and leaves scope for future studies. First, this study is limited to the context of China. In the future, the context of other countries can be included. Second, the study used the cross-sectional approach, but the longitudinal or mixed methodology could be adopted to examine the behavior of tourists more deeply. Third, in this study, TT has been examined as a mediator, but in the future, moderating variables (characteristics of tourists) could also be examined to study the relationship between independent and dependent variables.

## Data Availability Statement

The raw data supporting the conclusion of this article will be made available by the authors, without undue reservation.

## Ethics Statement

Ethical review and approval was not required for the study on human participants in accordance with the local legislation and institutional requirements. The patients/participants provided their written informed consent to participate in this study.

## Author Contributions

All authors listed have made a substantial, direct, and intellectual contribution to the work, and approved it for publication.

## Conflict of Interest

The authors declare that the research was conducted in the absence of any commercial or financial relationships that could be construed as a potential conflict of interest.

## Publisher’s Note

All claims expressed in this article are solely those of the authors and do not necessarily represent those of their affiliated organizations, or those of the publisher, the editors and the reviewers. Any product that may be evaluated in this article, or claim that may be made by its manufacturer, is not guaranteed or endorsed by the publisher.
